# Predicting the prognosis of epithelial ovarian cancer patients based on deep learning models

**DOI:** 10.3389/fonc.2025.1592746

**Published:** 2025-07-25

**Authors:** Zihan Li, Jiao Wang, Yixin Zhang, Zhen Yang, Fanchen Zhou, Xueting Bai, Qian Zhang, Wenchong Zhen, Rongxuan Xu, Wei Wu, Zhihan Yao, Xiaofeng Li, Yiming Yang

**Affiliations:** ^1^ Department of Epidemiology and Health Statistics, Dalian Medical University, Dalian, China; ^2^ Dalian Municipal Central Hospital, Central Hospital of Dalian University of Technology, Dalian, China

**Keywords:** deep learning, machine learning, epithelial ovarian cancer, prognosis, survival

## Abstract

**Background:**

Epithelial ovarian cancer(EOC) has a higher mortality and morbidity rate than other types, and it has a dramatic impact on the survival of ovarian cancer(OC) patients. Therefore, investigating, developing and validating prognostic models to predict overall survival(OS) in patients with epithelial ovarian cancer represents an area of research with significant clinical implications.

**Methods:**

Patients with a confirmed diagnosis of epithelial ovarian cancer from 2010 to 2017 in The Surveillance, Epidemiology, and End Results(SEER) database were identified for enrollment based on inclusion and exclusion criteria(N=10902). Patients with epithelial ovarian cancer diagnosed from 2010 to 2022 were selected from Dalian Municipal Central Hospital as an external validation cohort based on the same criteria (N=116). COX proportional risk regression for screening independent prognostic factors. Survival outcomes were compared between different risk subgroups based on Kaplan-Meier analysis. Three predictive models were developed using machine learning(ML) techniques, and another was a nomogram based on COX proportional risk regression for estimating 3-year and 5-year overall survival in patients with epithelial ovarian cancer. Evaluation of several models based on multiple metrics including C-index, ROC curve, calibration curve and decision curve analysis (DCA).

**Results:**

Through univariate and multivariate COX proportional risk regression analyses, we selected 12 significantly independent prognostic factors affecting overall survival (P<0.05). In conclusion, comparing several models cited, it was found that DeepSurv (Deep Survival) model had the best performance in both internal validation set and external validation set. The C-index for internal validation was 0.715, and the 3-year and 5-year ROC curves were 0.746 and 0.766; the C-index for external validation was 0.672, and the 3-year and 5-year ROC curves were 0.731 and 0.756.

**Conclusion:**

This study successfully developed a nomogram and three machine learning models, which collectively served as important predictive instruments to support clinical decision making.

## 1 Introduction

Ovarian cancer, as the fourth leading cause of cancer-related deaths in women (1), is also the fifth leading cause of death after several other cancers such as lung and breast cancer (2). The histologic types of ovarian cancer are classified by the World Health Organization(WHO) into the following broad categories:epithelial, germ cell, gonadal mesenchymal, metastatic and others (3). Among these histological subtypes, epithelial ovarian cancer, accounting for the majority of all types, and demonstrates distinct histopathological features (4). Epithelial ovarian cancer is complex and can be subdivided into five histological subtypes, including high-grade serous OC(HGSOC), low-grade serous OC(LGSOC), clear cell OC (CCOC), endometrioid OC (EMOC) and mucinous OC (MCOC),among which HGSOC and LGSOC belong to serous ovarian cancer (SOC). Different histological subtypes are characterized by different clinical characteristics and molecular profiles. According to a prospective study, ovarian cancer patients with different histological subtypes were diagnosed at different stages according to The International Federation of Gynecology and Obstetrics (FIGO) staging system (5). Studies have shown that different molecular subtypes can result in different prognostic outcomes (6, 7). This emphasizes the importance of considering histological subtypes in the prognosis of OC patients (8). Over the past 30 years, the five year relative survival rate for all cancers has a significant improvement, increasing by 20%. However,despite recent diagnostic and therapeutic advancements. Even in resource-rich countries such as the United States, the 5-year survival rate among ovarian cancer patients is merely 47% (9). The common treatments for ovarian cancer today are surgery and chemotherapy treatments (10). The clinical management of epithelial ovarian cancer faces great challenges, mainly due to the lack of reliable early diagnostic symptoms and effective diagnostic indicators. This diagnostic limitation leads to 70-75% of epithelial ovarian cancer cases being identified at advanced stages, seriously affecting patients survival and posing substantial threats to women’s health (11). Meantime, the advanced-stage diagnosis of patients often leads to patients missing the optimal time for surgery. Furthermore, even when surgical treatment is implemented, the efficacy of radical surgery is less effective, ultimately contributing to the poor survival in epithelial ovarian cancer patients (12). Although advanced medical techniques and drug therapies have been adopted for patients, the 5-year survival rate of patients is still less than 50% (13). Every year, about 230000 people are diagnosed with epithelial ovarian cancer, resulting in 150000 deaths (14).

Currently, the application of machine learning models in predicting the prognosis of ovarian cancer remains relatively rare. Firstly, although previous studies have used six machine learning methods to predict the survival rate of ovarian cancer (15), and other studies have used ten machine learning methods to predict the impact of preoperative blood characteristics on the prognosis of ovarian cancer (16). However, there are relatively fewer studies on prediction based on deep learning(DL) models, and potential reasons for this phenomenon may be that deep learning is currently more widely applied in research on image recognition (17–19). Moreover, most existing studies have generally not performed external validation of cited models (3, 13). Therefore, it is crucial to find effective methods to predict the prognosis of patients with epithelial ovarian cancer. Our study not only applied deep learning-based models such as DeepSurv and DeepHit for models establishment and evaluation but also incorporated external validation data to enhance the clinical utility of the results.

Nomogram is a sophisticated tool that can be used to assess survival with great predictive accuracy compared to conventional staging systems (20). The perceptual machine model, which is the basis for the development of modern neural networks, was created by Rosenblatt in 1958 (21). Deep learning is a novel machine learning (ML) technique. It has advantages over other machine learning methods such as logistic regression in solving complex computational problems (22). Some event-time machine learning models such as DeepSurv, DeepHit and Random Survival Forest (RSF) and so on, have been demonstrated to have good prediction performance (23), but it is unclear how these models predict the prognosis of epithelial ovarian cancer.

The Surveillance, Epidemiology, and End Results database, established in the United States, is an official cancer database. It contains population-based clinical survival data from registries for 34.6% of the national population (24). Deep learning is an algorithm based on neural network. In our study, we constructed survival analysis models for predicting patients with epithelial ovarian cancer by applying data from the SEER database. We developed a nomogram and implemented three machine learning models (DeepSurv, DeepHit, RSF), while simultaneously identifying critical prognostic factors in epithelial ovarian cancer patients. This provides clinicians with a powerful tool for accurate prognostic prediction and individualized risk assessment when treating patients with epithelial ovarian cancer.

## 2 Materials and methods

### 2.1 Data sources

The SEER database is supported by the National Cancer Institute (NCI) and has been in existence since 1973 to the present. Patients diagnosed with ovarian cancer between 2010 and 2017 were identified from the SEER*Stat software (version 8.4.3, https://seer.cancer.gov/seerstat/) (25). These data were publicly accessible and do not require ethics committee review or approval and informed patient consent (2). The primary tumor site code was C56.9-Ovarian. Patients with epithelial ovarian cancer were selected according to the inclusion and exclusion criteria, the International Classification of Diseases of Oncology ICD-O-3 Morphologic codes “8441/3-8442/3,8460/3-8463/3,9014/3” were used to identify patients with serous ovarian cancer; “8470/3-8472,8480/3- 8482/3,9015/3” for identifying women with mucinous ovarian cancer; “8380/3-8382/3,8560/3,8570/3” for identifying patients with endometrioid/adenocarcinoma; “8310/3, 8313/3” for identifying women with clear cell ovarian cancer. Patients in the SEER database were randomly assigned to both the training cohort and the internal validation cohort in a 7:3 ratio. In addition, we collected data on epithelial ovarian cancer patients who met the same criteria from 2010 to 2022 from Dalian Municipal Central Hospital as an external validation cohort. All clinical information was anonymized before analysis, and the study was approved by the Medical Research Ethics Committee of Dalian Municipal Central Hospital (Ethics Approval No. YN2024-111-01).

### 2.2 Selection of enrolled cases

Inclusion criteria: (1) Patients were diagnosed with epithelial ovarian cancer by pathologic diagnosis. (2) Patients were stage I-IV according to FIGO stage. (3) All follow-up and clinical data of the patients were available. (4) The patients underwent surgery at the primary site.

Exclusion criteria: (1) The patient’s histologic type was non epithelial ovarian cancer. (2) Combination of malignant tumors in other sites. (3) Patients with incomplete follow-up time. (4) Missing clinical or follow-up data. (5) Only be proven at autopsy or death.

### 2.3 Data collection and processing

#### 2.3.1 Data collection

We collected the following information on epithelial ovarian cancer patients in the SEER database: age at diagnosis, race, region of residence, marital status, serum CA125 level, surgery, tumor grade, histologic type, FIGO stage, T stage, N stage, tumor laterality, chemotherapy, bone metastasis, liver metastasis, brain metastasis, lung metastasis, survival time, survival status.

The following information on all enrolled patients was collected from Dalian Municipal Central Hospital: age at diagnosis, serum CA125 level, surgery, tumor grade, histologic type, FIGO stage, T stage, tumor laterality, chemotherapy, bone metastasis, liver metastasis, lung metastasis, survival time, survival status.

#### 2.3.2 Data processing

Samples with missing data in the SEER database as well as in the clinical data were excluded from this study. Statistically significant variables(P<0.05) were identified by univariate and multivariate COX proportional risk regression, and the results of multivariate regression screening were included in the models as pretreatment variables for prediction.

### 2.4 Survival analysis model

In order to select meaningful indicators, we first conducted a univariate analysis based on COX proportional risk regression models (P<0.05). Meaningful indicators were then incorporated into the multifactorial analysis, not only to create a nomogram but also to incorporate them into survival analysis modes. The three machine learning models used in this study to perform survival analysis include the decision tree-based model Random Survival Forest (RSF) (26) and two deep learning based models [DeepHit (27) and DeepSurv (28)]. DeepSurv, a deep neural network framework built upon the Cox proportional hazards model. This method outperforms linear and nonlinear survival analysis methods in predicting patients risk. DeepHit represents a deep learning-based non-proportional hazards algorithm that employs multi-task learning to address competing events (23). RSF is a decision tree-based machine learning method for survival analysis. It can efficiently handle nonlinear effects, correlation parameters and variable interactions (29).

### 2.5 Statistical analysis

The patients data in this study were statistically analyzed using R software version 4.4.3 and Excel. Age was classified as young (<45 years), middle-aged (45–59 years), and old (≥60 years) according to the World Health Organization population age distribution. Categorical variables were expressed as counts and percentages, and baseline characteristics of the training and testing cohorts were compared using chi-square and Fisher’s exact tests. Comparison of survival rates in different risk groups of patients with epithelial ovarian cancer was based on Kaplan-Meier survival curves by R-studio 4.4.3 software. And the survival rates of the different groups were analyzed using the log-rank test. The rms, foreign, and survival packages in R software were used to create the nomogram, and we used the pyCOX package in python 3.9.0 to build the DeepSurv and DeepHit models, and the RandomForestSRC package in R software was used to build the RSF model. The calibration curves allowed assessment of the relationship between patients follow-up outcomes and predicted survival. Calibration curves were plotted using the rms R software package for assessing the calibration of the cited models, and Bootstrap methods were applied for repeat sampling (B =1000); ROC curves were plotted over time based on the use of the timeROC R package; and DCA decision curves were plotted using the ggDCA package for nomogram and different machine learning models (30, 31).

## 3 Results

### 3.1 Data on demographic and clinical characteristics

**Figure 1:**
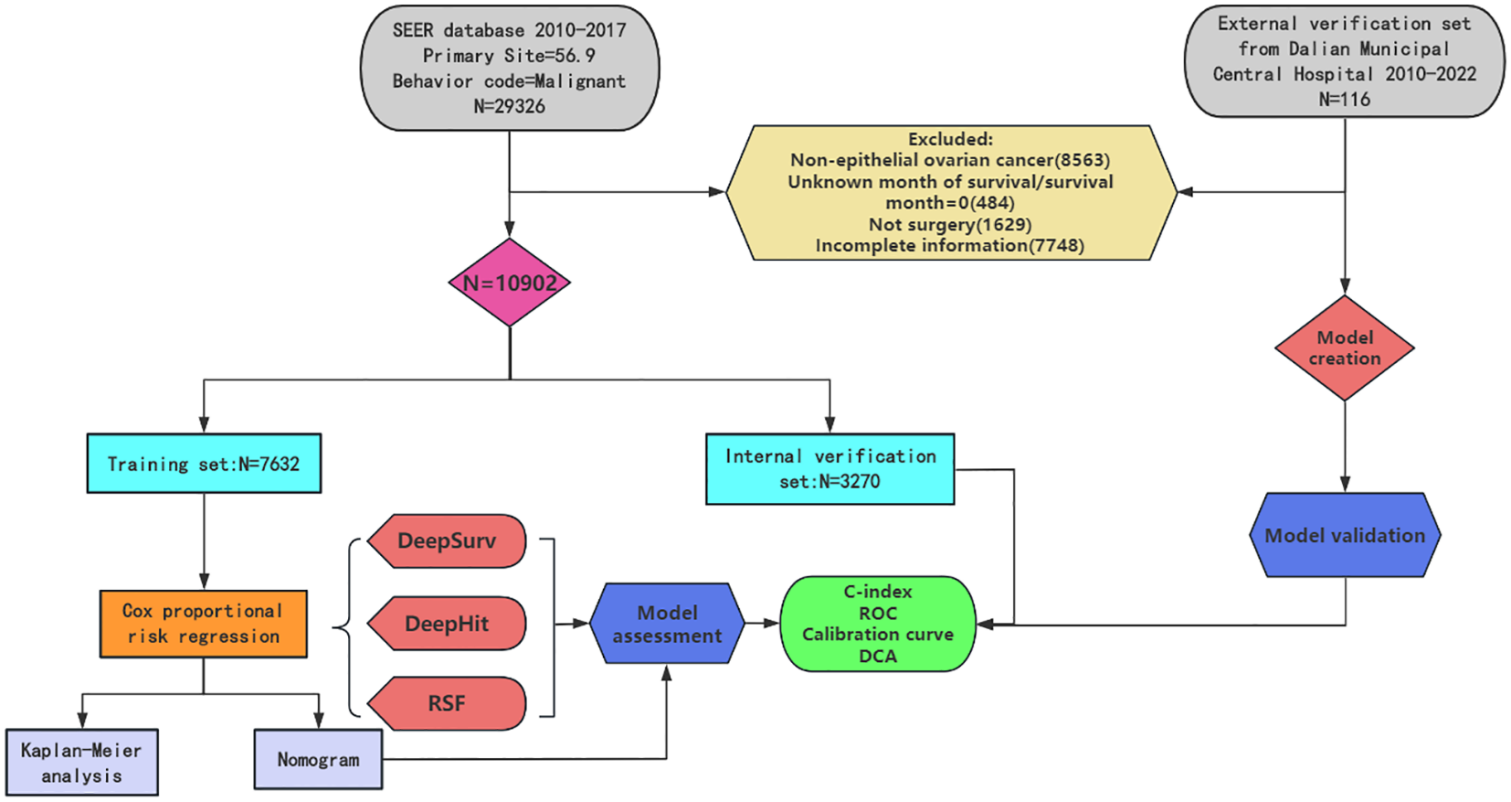
The flowchart and patients selection process for this study.

The demographic and clinical characteristics of the 10902 patients with primary epithelial ovarian cancer in the SEER database are presented in **Table 1**, which showed that 7632 patients were assigned to the training cohort and 3270 patients to the internal validation cohort by R-studio 4.4.3 software. There were no significant differences between the two cohorts in terms of demographic and clinicopathologic characteristics (P>0.05), which represents comparable data between the two cohorts. The distribution of age groups revealed that the majority of epithelial ovarian cancer patients were older (49.8%), with a higher percentage of married patients (80.2%), a high percentage of serum CA125 positivity (87.9%), more serous ovarian cancer (70.3%), most of them grade III/IV (73.7%), and FIGOIII-IV (63.6%). Only a few patients did not receive chemotherapy (17.3%). The flowchart and patients selection process for this study was shown in **Figure 1**.

**Table 1:** General data on training and internal validation cohort for patients with epithelial ovarian cancer n (%).

Characteristics	Test (N=3270)	Train (N=7632)	Overall (N=10902)	P
Age				0.796
<45	370 (11.3%)	867 (11.4%)	1237 (11.3%)	
≥60	1644 (50.3%)	3785 (49.6%)	5429 (49.8%)	
45-59	1256 (38.4%)	2980 (39.0%)	4236 (38.9%)	
Rural/Urban				0.849
metropolitan	2932 (89.7%)	6854 (89.8%)	9786 (89.8%)	
nonmetropolitan	338 (10.3%)	778 (10.2%)	1116 (10.2%)	
Race				0.693
American Indian/Alaska Native	25 (0.8%)	52 (0.7%)	77 (0.7%)	
Asian or Pacific Islander	345 (10.6%)	858 (11.2%)	1203 (11.0%)	
Black	205 (6.3%)	461 (6.0%)	666 (6.1%)	
White	2695 (82.4%)	6261 (82.0%)	8956 (82.2%)	
Grade				0.304
GradeI/II	839 (25.7%)	2032 (26.6%)	2871 (26.3%)	
GradeIII/IV	2431 (74.3%)	5600 (73.4%)	8031 (73.7%)	
Histologic type				0.802
Clear	261 (8.0%)	614 (8.0%)	875 (8.0%)	
Endo/adeno	453 (13.9%)	1079 (14.1%)	1532 (14.1%)	
Mucinous	262 (8.0%)	571 (7.5%)	833 (7.6%)	
Serous	2294 (70.2%)	5368 (70.3%)	7662 (70.3%)	
FIGO				0.268
I/II	1215 (37.2%)	2749 (36.0%)	3964 (36.4%)	
III/IV	2055 (62.8%)	4883 (64.0%)	6938 (63.6%)	
Surgery				0.876
Debulking	1637 (50.1%)	3840 (50.3%)	5477 (50.2%)	
Local resection	1557 (47.6%)	3626 (47.5%)	5183 (47.5%)	
Pelvic exenteration	76 (2.3%)	166 (2.2%)	242 (2.2%)	
Chemotherapy				0.373
No/Unknown	582 (17.8%)	1303 (17.1%)	1885 (17.3%)	
Yes	2688 (82.2%)	6329 (82.9%)	9017 (82.7%)	
T stage				0.612
T1-T2	1370 (41.9%)	3156 (41.4%)	4526 (41.5%)	
T3	1900 (58.1%)	4476 (58.6%)	6376 (58.5%)	
CA125				0.002
Negative	446 (13.6%)	878 (11.5%)	1324 (12.1%)	
Positive	2824 (86.4%)	6754 (88.5%)	9578 (87.9%)	
N stage				0.029
N0	2464 (75.4%)	5596 (73.3%)	8060 (73.9%)	
N1	806 (24.6%)	2036 (26.7%)	2842 (26.1%)	
Laterality				0.392
Bilateral	1427 (43.6%)	3400 (44.5%)	4827 (44.3%)	
Unilateral	1843 (56.4%)	4232 (55.5%)	6075 (55.7%)	
Bone				0.842
No	3262 (99.8%)	7610 (99.7%)	10872 (99.7%)	
Yes	8 (0.2%)	22 (0.3%)	30 (0.3%)	
Brain				1.000
No	3268 (99.9%)	7629 (100.0%)	10897 (100.0%)	
Yes	2 (0.1%)	3 (0.0%)	5 (0.0%)	
Lung				0.525
No	3165 (96.8%)	7406 (97.0%)	10571 (97.0%)	
Yes	105 (3.2%)	226 (3.0%)	331 (3.0%)	
Liver				0.156
No	3159 (96.6%)	7328 (96.0%)	10487 (96.2%)	
Yes	111 (3.4%)	304 (4.0%)	415 (3.8%)	
Marital status				0.593
Married	2634 (80.6%)	6112 (80.1%)	8746 (80.2%)	
Not married	636 (19.4%)	1520 (19.9%)	2156 (19.8%)	

### 3.2 Survival analysis

First, we performed univariate COX proportional risk regression analysis on data from the training cohort of patients with epithelial ovarian cancer to identify prognostic variables. And the results showed that age at diagnosis, serum CA125 level, surgery, tumor grade, histologic type, FIGO stage, T stage, N stage, tumor laterality, chemotherapy, marital status, and bone metastasis, liver metastasis, and lung metastasis were the risk factors (P<0.05). Subsequent multifactorial COX proportional risk regression analysis showed that age at diagnosis, serum CA125 level, surgery, tumor grade, histologic type, FIGO stage, T stage, tumor laterality, chemotherapy, and bone metastasis, liver metastasis, and lung metastasis were independent prognostic factors affecting the overall survival of patients (P < 0.05). The results of the univariate and multivariate COX proportional risk regressions were displayed in the **Table 2**.

**Table 2:** Epithelial ovarian cancer patients performed univariate and multivariate COX proportional risk regression analyses on the training cohort of patients.

Characteristics	Univariate analysis HR (95%CI)	P	Multivariate analysis HR (95%CI)	P
Age				
<45	Ref		Ref	
≥60	2.57 (2.26-2.92)	<0.001	1.87 (1.64-2.13)	<0.001
45-59	1.59 (1.40-1.82)	<0.001	1.31 (1.14-1.50)	<0.001
Rural/Urban				
metropolitan	Ref			
nonmetropolitan	1.04 (0.94-1.15)	0.415		
Race				
American Indian/Alaska Native	Ref			
Asian or Pacific Islander	0.77 (0.52-1.13)	0.180		
Black	1.33 (0.90-1.96)	0.152		
White	0.97 (0.67-1.40)	0.857		
Grade				
GradeI/II	Ref		Ref	
GradeIII/IV	2.90 (2.65-3.17)	<0.001	1.49 (1.34-1.65)	<0.001
Histologic type				
Clear	Ref		Ref	
Endo/adeno	0.49 (0.41-0.59)	<0.001	0.55 (0.45-0.66)	<0.001
Mucinous	0.65 (0.52-0.80)	<0.001	1.08 (0.86-1.35)	0.531
Serous	2.01 (1.76-2.31)	<0.001	0.70 (0.60-0.81)	<0.001
FIGO				
I-II	Ref		Ref	
III-IV	5.15 (4.70-5.63)	<0.001	2.30 (1.92-2.76)	<0.001
Surgery				
Debulking	Ref		Ref	
Local resection	0.44 (0.41-0.47)	<0.001	0.88 (0.82-0.94)	<0.001
Pelvic exenteration	1.02 (0.84-1.23)	0.872	0.90 (0.74-1.09),	0.266
Chemotherapy				
No/Unknown	Ref		Ref	
Yes	1.80 (1.63-1.99)	<0.001	0.75 (0.67-0.83)	<0.001
T stage				
T1-T2	Ref		Ref	
T3	4.54 (4.19-4.92)	<0.001	1.74 (1.50-2.03)	<0.001
CA125				
Negative	Ref		Ref	
Positive	2.96 (2.57-3.40)	<0.001	1.45 (1.25-1.68)	<0.001
N stage				
N0	Ref		Ref	
N1	1.80 (1.69-1.93)	<0.001	1.04 (0.97-1.12)	0.253
Laterality				
Bilateral	Ref		Ref	
Unilateral	0.48 (0.45-0.51)	<0.001	0.85 (0.79-0.91)	<0.001
Bone				
No	Ref		Ref	
Yes	4.02 (2.59-6.23)	<0.001	1.81 (1.16-2.83)	0.009
Brain				
No	Ref			
Yes	1.42 (0.36-5.69)	0.618		
Lung				
No	Ref		Ref	
Yes	2.76 (2.38-3.19)	<0.001	1.59 (1.37-1.84)	<0.001
Liver				
No	Ref		Ref	
Yes	2.28 (2.00-2.60)	<0.001	1.35 (1.18-1.54)	<0.001
Marital status				
Married	Ref		Ref	
Not married	0.84 (0.78-0.91)	<0.001	1.09 (1.00-1.18)	0.051

In addition, we used Kaplan-Meier analysis to assess 14 variables screened by univariate Cox risk regression in the training cohort. In the meantime, we performed Kaplan-Meier analysis of these 14 factors in our internal validation cohort. And the results were all shown in the **Supplementary Material**, with corresponding results for the training cohort and internal validation cohort designated in the **Supplementary Figures 7** and **8**.

### 3.3 Creation of nomogram

**Figure 2:**
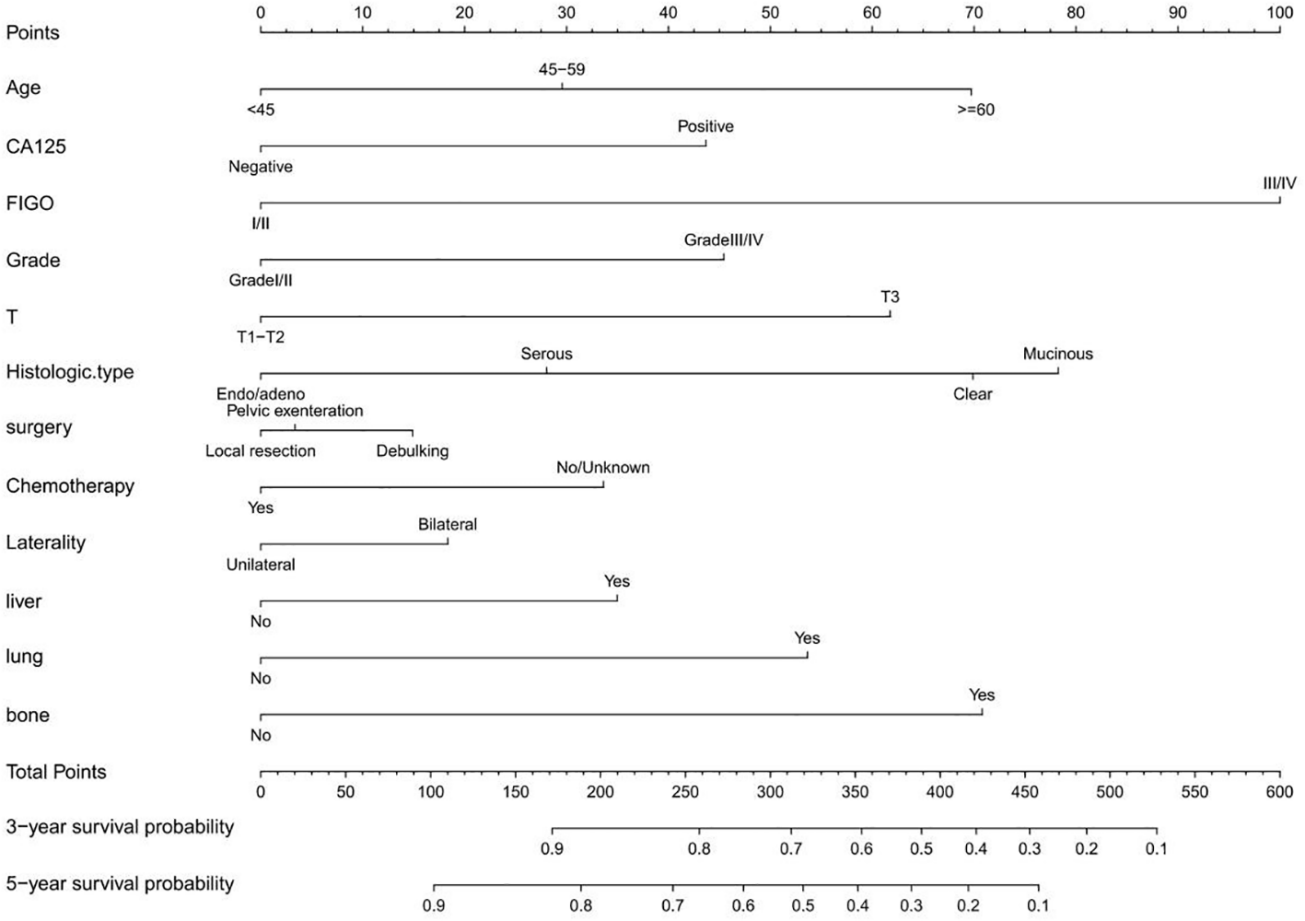
Nomogram of 3 year and 5 year survival prediction for patients with epithelial ovarian cancer.

Variables screened in the multifactorial COX proportional risk regression(P < 0.05) were analyzed by applying R software to create a nomogram, and the result was presented in **Figure 2**. The nomogram was constructed based on COX proportional risk regression results. The results showed that the occurrence of FIGO stage contributed most to the nomogram, followed by histologic type. From our established nomogram, we found that patients with FIGO stage = III/IV had a higher risk of death than patients with FIGO stage of I/II, which was consistent with the finding that patients with advanced ovarian cancer have a worse prognosis. Risk scores were determined based on individual scores calculated using nomogram (32).

### 3.4 Validation of nomogram

**Figure 3:**
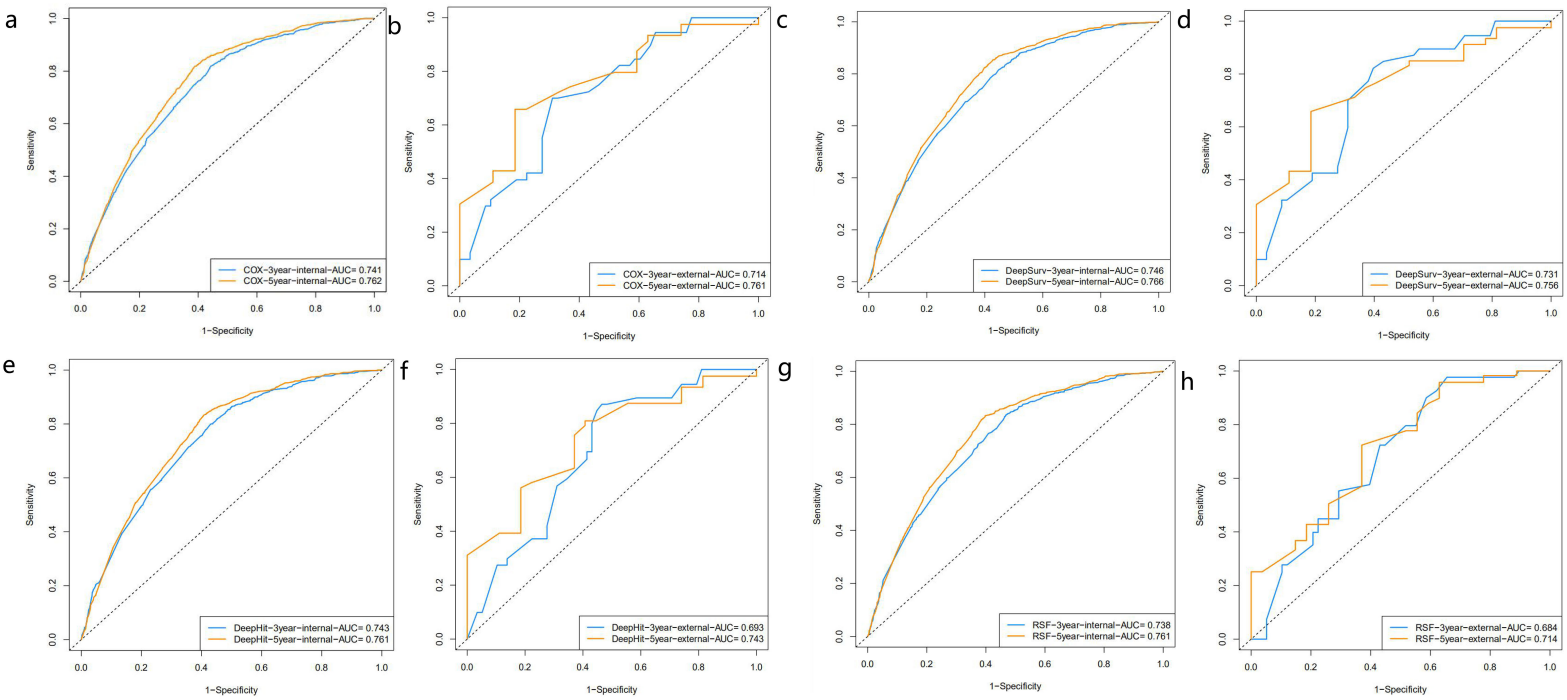
ROC curves of 3 year and 5 year survival prediction in patients with epithelial ovarian cancer [**(a, c, e, g)** are the internal validation cohort, **(b, d, f, h)** are the external validation cohort].

**Figure 4:**
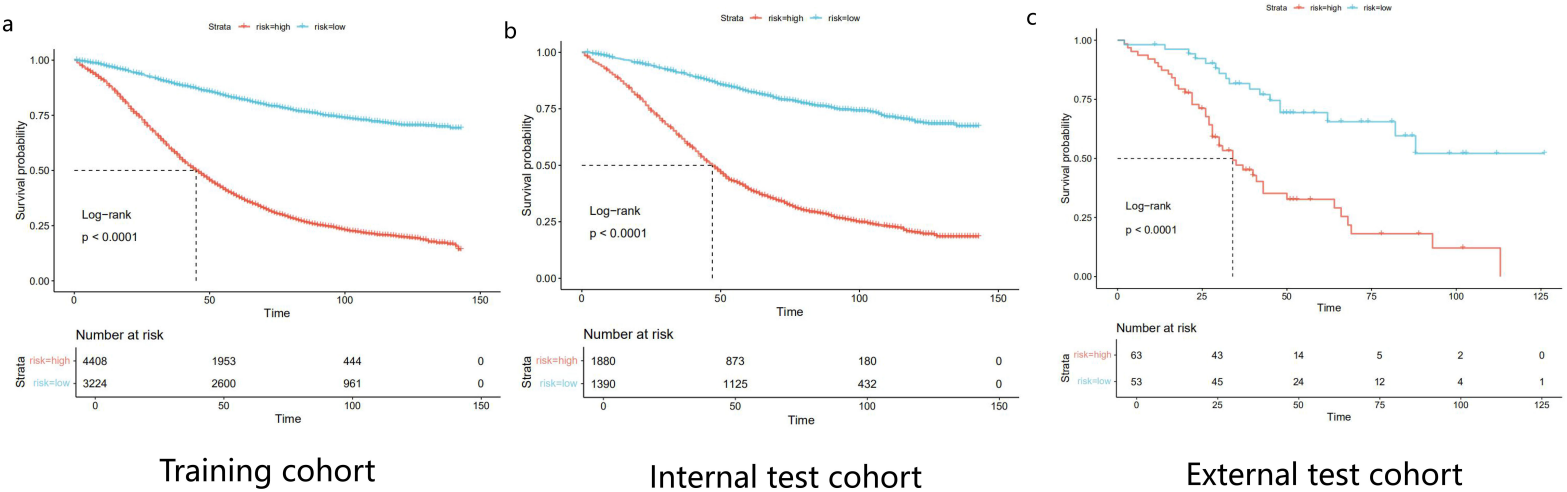
Kaplan-Meier survival curves comparing overall survival in patients with epithelial ovarian cancer in different risk groups. **(a)** Training cohort. **(b)** Internal test cohort. **(c)** External test cohort.

We utilized the internal validation cohort of the SEER database to validate the model built based on the training cohort, and the area under the ROC curve was used to evaluate the accuracy of the model in **Figure 3**. Calibration curves and DCA curves were showed in the **Supplementary Figures 9** and **10** to assess the calibration and clinical utility of the model at 3-year and 5-year,respectively. The results of the study found that the C-index of the COX proportional risk regression model for the internal validation cohort and the external validation cohort were 0.711 and 0.664,respectively. Epithelial ovarian cancer patients from Dalian Municipal Central Hospital (N=116) were used as external validation, and the variable characteristics of the patients were shown in **Table 3**. Based on nomogram score, patients were categorized into high and low risk groups. According to **Figure 4** survival curves plotted based on Kaplan-Meier analysis showed a significant difference in survival between the low and high risk groups (p<0.0001). Patients in the high risk group had significantly worse survival outcomes compared to low risk patients.

**Table 3:** General data on external validation cohort for patients with epithelial ovarian cancer n (%).

Characteristics	Test (N=116)
Age	
<45	9 (7.8%)
≥60	56 (48.3%)
45-59	51 (44.0%)
Grade	
Grade I/II	4 (3.4%)
Grade III/IV	112 (96.6%)
Histologic type	
Clear	7 (6.0%)
Endo/adeno	5 (4.3%)
Mucinous	3 (2.6%)
Serous	101 (87.1%)
FIGO	
I/II	5 (4.3%)
III/IV	111 (95.7%)
Surgery	
Debulking	55 (47.4%)
Local resection	45 (38.8%)
Pelvic exenteration	16 (13.8%)
Chemotherapy	
No/Unknown	77 (66.4%)
Yes	39 (33.6%)
T stage	
T1-T2	5 (4.3%)
T3	111 (95.7%)
CA125	
Negative	8 (6.9%)
Positive	108 (93.1%)
Laterality	
Bilateral	105 (90.5%)
Unilateral	11 (9.5%)
Bone	
No	115 (99.1%)
Yes	1 (0.9%)
Lung	
No	115 (99.1%)
Yes	1 (0.9%)
Liver	
No	114 (98.3%)
Yes	2 (1.7%)

### 3.5 Comparison of model performance

We constructed three machine learning models for survival analysis using the training cohort. Harrell’s C-index was first used to measure the relationship between the model’s prediction of the risk profile and the actual survival of the patients to reflect the model’s predictive effectiveness. In the DeepSurv model, the C-index of the internal validation cohort was 0.715 and the external validation cohort was 0.672, whereas the C-index of the internal validation cohort of the DeepHit model was 0.712 and the external validation cohort was 0.661. The C-index of the internal validation set and the C-index of the external validation set of the RSF model were 0.709 and 0.634, respectively.

**Figure 5:**
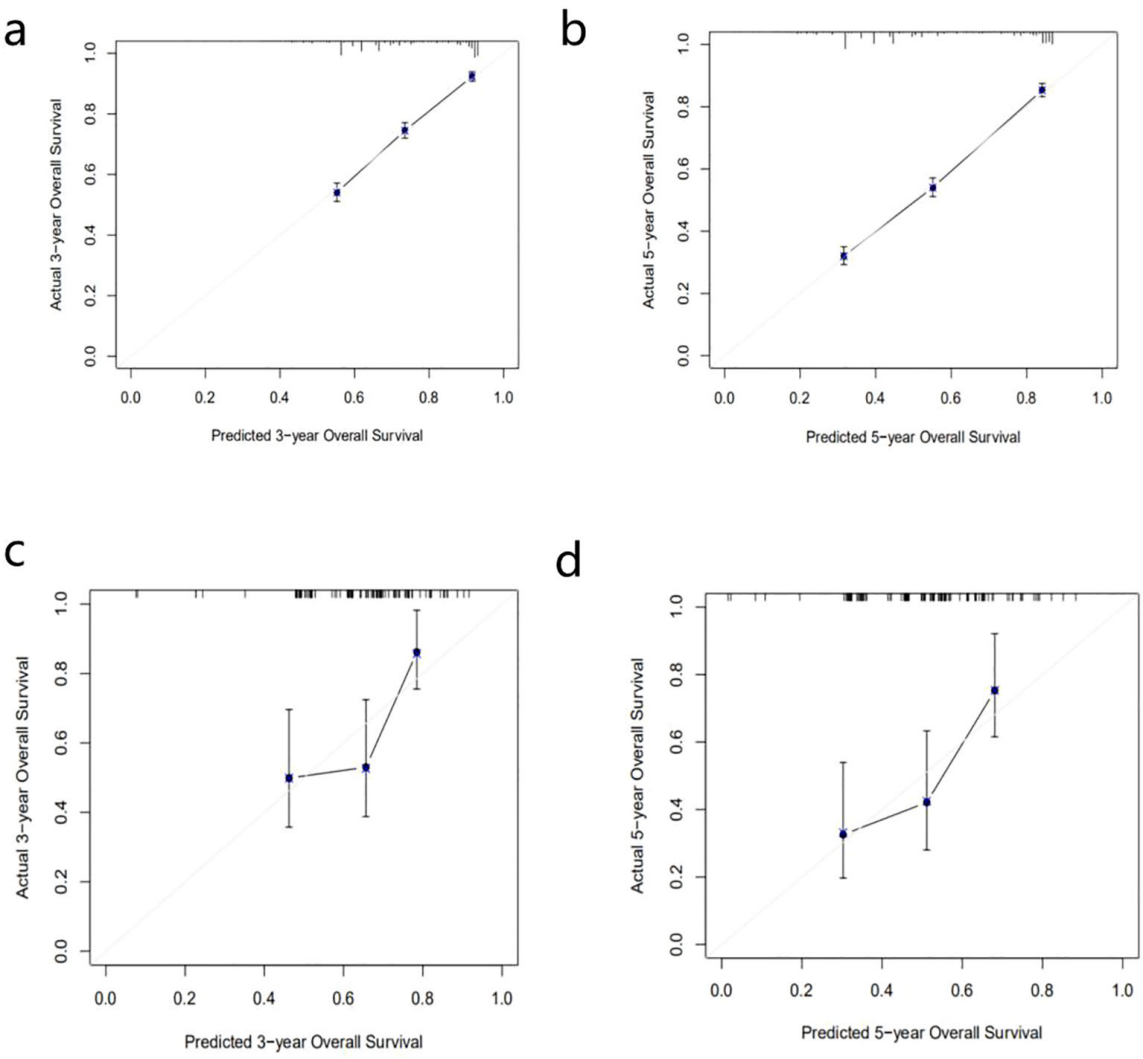
DeepSurv 3 year and 5 year survival calibration curves for patients with epithelial ovarian cancer **(a, b)** are internal validation set; **(c, d)** are external validation set).

**Figure 6:**
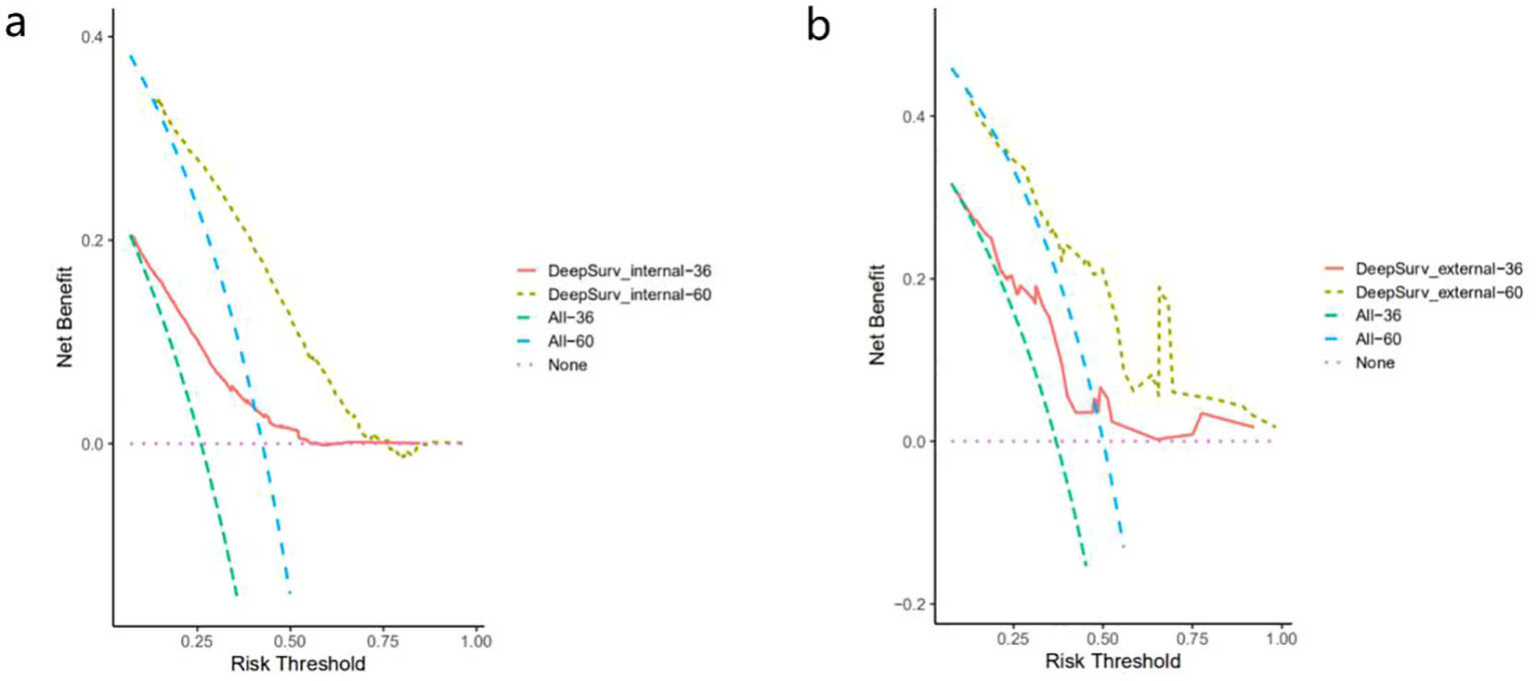
DeepSurv 3 year and 5 year decision curve analysis for patients with epithelial ovarian cancer. **(a)** internal validation sex; **(b)** external validation set.

We then calculated 3-year and 5-year AUC values for the three machine learning models to verify the accuracy of the models. **Figure 3** showed the ROC curves assessing different survival analysis models for predicting patients survival outcomes at three and five years, representing the overall performance of the models. We also validated these models by applying calibration curves and DCA decision curves. DeepSurv presented superior performance in our study. Its calibration curves for internal and external validation were displayed in **Figure 5**. The calibration curves for the other models were displayed in **Supplementary Figure 9**. The results showed that the RSF model had the best calibration, and the DeepHit model was slightly less calibrated than other models. The analysis results of the DCA decision curve of the DeepSurv model were shown in **Figure 6**, and the DCA decision curve results of the other models were presented in **Supplementary Figure 10**. If the net benefit rate of the curve is higher than the extreme curve, it indicates that the curve has some application. The results founded that the internal validation results are better than the external validation results. Our study revealed that the COX proportional risk regression model, which is a traditional model, performed similarly to the other machine learning models. However, in the results in **Table 4** we found that the DeepSurv model outperformed the other machine learning models as well as the COX proportional risk regression model in both the internal and the external validation. Integrative analysis of these assessment metrics conclusively found that DeepSurv model outperform other models in both internal and external validation cohorts. **Table 4** showed a comparison of the C-index and AUC values for several models.

**Table 4:** COX proportional risk regression model and three machine learning models C-index and AUC values.

Model	COX	DeepSurv	DeepHit	RSF
Internal verification C-index	0.711	0.715	0.712	0.709
External verification C-index	0.664	0.672	0.661	0.634
Internal verification 3-yearAUC	0.741	0.746	0.743	0.738
Internal verification 5-yearAUC	0.762	0.766	0.761	0.761
External verification 3-yearAUC	0.714	0.731	0.693	0.684
External verification 5-yearAUC	0.761	0.756	0.743	0.714

## 4 Discussion

Among the principal gynecologic cancers (endometrial, cervical, and ovarian cancer), ovarian cancer has the second highest mortality rate and the worst prognosis (4), and represents a significant clinical challenge in women’s health. Ovarian cancer is classified based on histologic type, and epithelial ovarian cancer is arguably the most common type (33). The symptoms of early stage ovarian cancer are relatively less obvious. And the prognosis of patients relatively display favorable prognosis. However, about 75% of ovarian cancer patients are advanced at the time of diagnosis. Due to the lack of early specific symptoms and effective screening tools in these patients, the diagnosis of ovarian cancer can be delayed. This delay in diagnosis seriously affects the prognosis of patients and is a key factor in the generally poor prognosis of ovarian cancer (34). Therefore, more appropriate methods should be explored to deeply investigate the risk factors affecting the prognosis of patients with epithelial ovarian cancer. In our study, the best model we cited, DeepSurv, we found that its 3-year and 5-year AUC values in internal validation were 0.746,0.766; its AUC values in external validation were 0.731 and 0.756. It reflected that the model had relatively good accuracy.

Based on real clinical studies, DeepSurv demonstrated equivalent or better performance than other survival analysis methods in terms of time-to-event data. Our results showed that the overall performance of the DeepSurv model was superior to that of the traditional Cox proportional hazards regression model. The results demonstrated that the DeepSurv model outperformed traditional methods in complex survival analysis, particularly with high-dimensional data or non-proportional hazards. This advantage may derive from DeepSurv’s ability to jointly consider both linear and nonlinear effects of covariates. Beyond prognostic prediction, DeepSurv could also be used in survival analysis to generate personalized treatment recommendations based on the predictive impact of treatment regimens on individual risks, which highlighted an important direction for further research to better leverage the potential of this model (28, 35).

Numerous clinical studies have consistently indicated that surgical intervention significantly influences patients survival, with non-surgical treatment associated with much worse prognosis. Furthermore, the surgical approach significantly influences patients survival outcomes (13, 36, 37). Studies have shown that residual lesions following debulking surgery are an important prognostic factor affecting the overall survival rate of patients. Alternatively, failure to achieve satisfactory debulking may be the reason for the poor prognosis of patients after debulking surgery (38, 39). Zheng et al. conducted an analysis of surgical outcomes, revealing that patients undergoing pelvic exenteration demonstrated significantly higher mortality risk compared to other surgical approaches (30). According to the study conducted by Song et al, advanced stage (III/IV) epithelial ovarian cancer patients undergoing debulking or pelvic exenteration demonstrated significantly improved survival outcomes, with reduced mortality risk (36). Cheng et al. demonstrated significantly improved survival outcomes in patients undergoing local resection (13). We found that our study was consistent with the findings of Cheng et al.

Role of chemotherapy in ovarian cancer still controversial. Whether in studies of different histological types such as mucinous cancer, clear cell cancer, or older epithelial ovarian cancer based on age group classification (13, 40, 41). In our study, according to the COX proportional risk regression analysis, it can be determined that whether or not patients undergoes chemotherapy is a significant risk factor influencing the overall survival of patients with epithelial ovarian cancer (P < 0.05), the nomogram revealed that patients with unknown chemotherapy status or those who did not receive chemotherapy demonstrated significantly higher mortality. The International Federation of Gynecology and Obstetrics (FIGO) clinical data reveal that advanced stage (III-IV) ovarian cancer patients who receive neoadjuvant chemotherapy may survive better than those who do not (42). A prior randomized trial demonstrated that hyperthermic intraperitoneal chemotherapy (HIPEC) combined with interstitial cytoreductive surgery and neoadjuvant chemotherapy is a promising option for improving the 5-year overall survival and disease-free survival (DFS) of patients with primary ovarian cancer (43). At the same time, a retrospective analysis in Italy found that cytoreductive surgery (peritonectomy procedures) combined with HIPEC can treat advanced ovarian cancer (44). Pressurized intraperitoneal aerosol chemotherapy(PIPAC) therapy has demonstrated significant efficacy in the treatment of patients with peritoneal carcinomatosis, and it is one of the most innovative methods of intraperitoneal chemotherapy (45). However, since the only available data on chemotherapy in the SEER database is whether the patients received chemotherapy or not. And the surgical information does not include whether patients underwent peritonectomy. Therefore, it is difficult to analyze specifically the effects of neoadjuvant chemotherapy, PIPAC and HIPEC. However, we must acknowledge that these methods show promising survival benefits on the survival outcomes of ovarian cancer patients and are of great significance in the field of therapeutic community. In the future, we will focus on strengthening research on neoadjuvant chemotherapy and PIPAC, while also increasing our exploration of peritonectomy and HIPEC.

Histological type is strongly associated with the prognosis of ovarian cancer. Zhou et al. found that among epithelial ovarian cancer patients, mucinous ovarian cancer and clear cell cancer displayed significantly poorer survival compared to serous ovarian cancer. Meantime, better survival in endometrioid ovarian cancer compared to serous ovarian cancer (2). This finding has been validated by our study, further strengthening the clinical significance of histological type as a prognostic factor. This further demonstrated that different histological subtypes were closely related to patients prognosis, with different subtypes leading to different outcomes (8). Therefore, histological subtypes should receive further attention in studies on ovarian cancer prognosis.

We referenced the study by De Felice F et al., which evaluated focused on healthcare professionals’ awareness and adherence to evidence-based nutritional interventions, with an emphasis on the feasibility of clinical nutrition plans, implementation of nutritional assessment plans, composition of multidisciplinary teams and the proficient utilization of screening tools. Current research emphasizes the critical role of nutritional care in oncology (46). However, many patients do not receive adequate nutritional support during treatment, which inevitably increases the risk of weight loss, poor tolerance to treatment and treatment-related complications in cancer patients (47, 48). Therefore, providing adequate nutrition during hospitalization is critical to maintain energy balance for cancer patients. Through this initiative, thereby improving treatment outcomes and ultimately improving patients’ quality of life (49, 50).

FIGO stage is always an important prognostic factor for ovarian cancer patients. And studies have shown that the later the FIGO stage, the worse the prognosis (51). The same conclusion can be drawn from the nomogram we have created. The prognosis of many cancers is related to the age of the patients. With Zhou et al. demonstrating a strong correlation between older patients and poorer clinical outcomes (2). Our study revealed this age-related prognostic pattern, further validating the clinical significance of age stratification in cancer prognosis. In this study, we first constructed a nomogram and built multiple survival-related machine learning models for variables selected by COX proportional risk regression to predict overall survival in patients with epithelial ovarian cancer, and then we performed a Kaplan-Meier survival analysis on 14 variables identified by COX proportional risk regression. Eventually we found that: the closer the FIGO stage was to the advanced stage, mucinous ovarian cancer, older age, positive serum CA125 level, larger tumor (T stage=T3), the less differentiated the tumor, do not or unknown receive chemotherapy, occurrence of metastasis to organs such as liver, lung, bone, etc, and tumors at bilateral sites, the shorter the survival time and the worse the survival. The results of the Kaplan-Meier survival curve analysis verified that all of the factors that we had included were significant (P < 0.05).

Adeoye applied DeepSurv, DeepHit, RSF, and COX-Time simultaneously to survival prediction of oral cancer (52); Li et al. applied CPH, DeepSurv, DeepHit, RSF were applied to the survival prediction of myeloma (53); Yang et al. applied COX-Time, N-MTLR, GBM, DeepSurv, DeepHit, and RSF to the survival prediction of colorectal cancer (23). Reilly et al. analyzed and validated deep neural network algorithms for the detection of ovarian cancer and found that the application of deep neural network algorithms in biomarker detection lays the foundation for future research (54). Our study combines three ML techniques(DeepSurv, DeepHit, RSF) and COX proportional risk regression model to predict the prognosis of patients with epithelial ovarian cancer. By comparing the models performance we selected the best predictive model—DeepSurv. Our study not only validated the model using internal validation, but also collected clinical data for external validation, thereby greatly enhancing the generalizability and clinical applicability of the model. The results proved that DeepSurv model was the optimal model both in the internal and external validation cohort. DeepSurv has great potential to complement traditional survival analysis methods and contribute to the development of the healthcare industry, thereby enhancing predictive accuracy in complex clinical scenarios (28).

In summary, we provided a deep learning based model—DeepSurv, for predicting overall survival of patients with epithelial ovarian cancer, as it outperforms other models in both internal and external validation sets. Of course other models also have good predictive performance. And the results of this study can provide some reference and guidance for the future clinical development of better treatment strategies, which can improve the quality of survival and increase the survival rate of epithelial ovarian cancer patients.

However, some limitations remain in our study. Firstly, our study is a retrospective study, and some information bias is inevitable in the process of collecting external validation follow-up data. Secondly, the database lacks more detailed prognostic information (including specific surgical conditions and postoperative complications, molecular and genomic data), and the baseline table shows that the majority of our study population is white, which may lead to bias in subsequent analyses. DeepSurv is somewhat of a black box. Deep neural networks, characterized by their complex multi-layered nonlinear architecture. Due to their multi-layer non-linear structure, making their internal decision-making processes difficult to interpret (55). Challenges remain for us in understanding the computations or resulting limitations during the model construction process (56). Therefore, we will continue to update and supervise nomogram and survival analysis models in future research efforts. Through this to ensure their continued validity and generalizability for clinical studies.

## 5 Conclusion

In this study, we established a nomogram for predicting epithelial ovarian cancer prognosis, and also applied DeepSurv and DeepHit and RSF models to predict postoperative prognosis of patients with epithelial ovarian cancer for the first time, which provides some reference value for clinical practice.

## Data Availability

The original contributions presented in the study are included in the article/**Supplementary Material**. Further inquiries can be directed to the corresponding authors.
