# Supplementary material for: Predicting the prognosis of epithelial ovarian cancer patients based on deep learning models
Source: Front Oncol. 2025 Jul 25;15:1592746. doi: 10.3389/fonc.2025.1592746 (PMC12331489; doi:10.3389/fonc.2025.1592746)
Supplement: Supplementary file 1 [file DataSheet1.pdf]

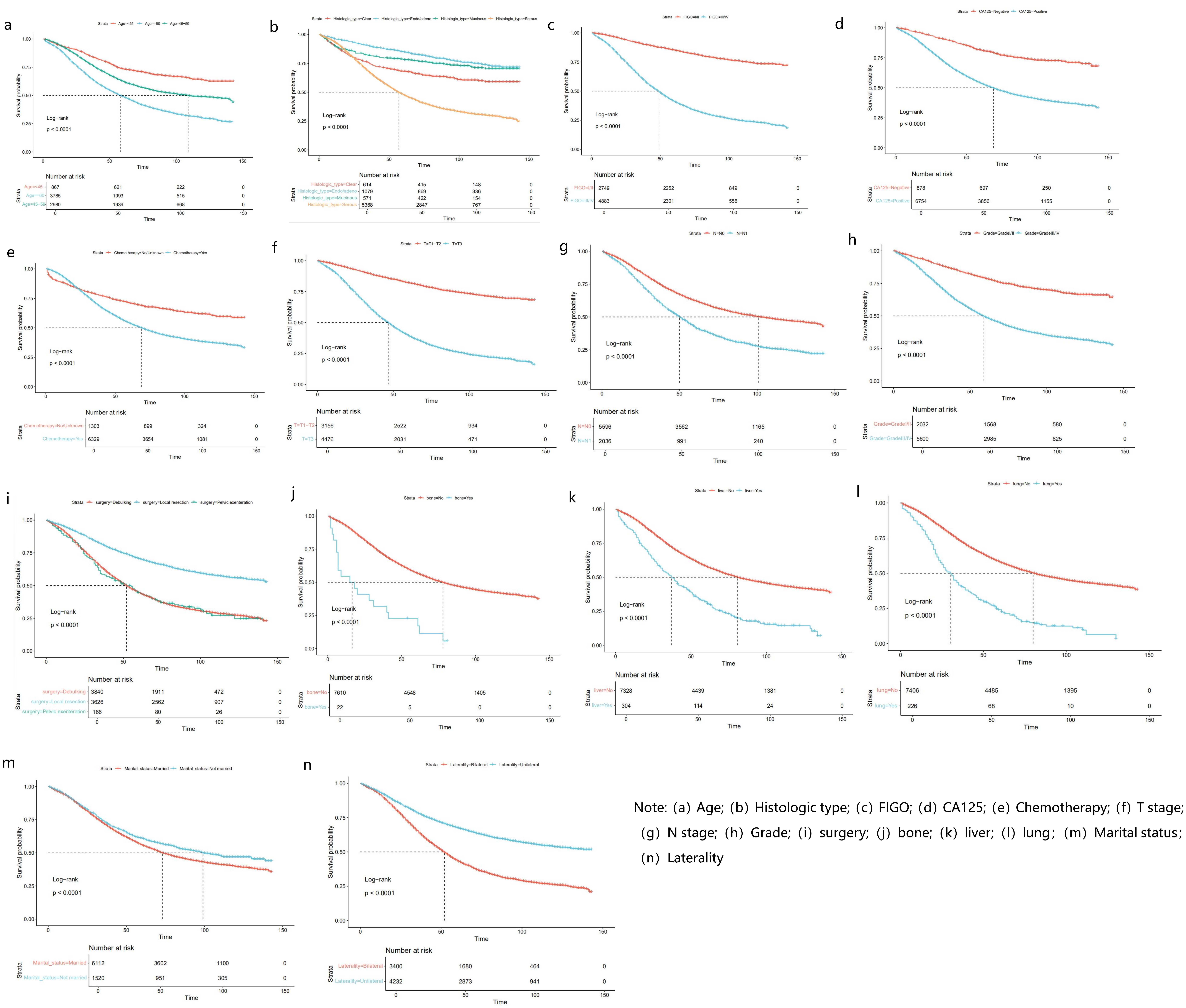

Note: (a) Age; (b) Histologic type; (c) FIGO; (d) CA125; (e) Chemotherapy; (f) T stage; (g) N stage; (h) Grade; (i) surgery; (j) bone; (k) liver; (l) lung; (m) Marital status; (n) Laterality
