# Supplementary material for: Predicting the prognosis of epithelial ovarian cancer patients based on deep learning models
Source: Front Oncol. 2025 Jul 25;15:1592746. doi: 10.3389/fonc.2025.1592746 (PMC12331489; doi:10.3389/fonc.2025.1592746)
Supplement: Supplementary file 3 [file DataSheet3.pdf]

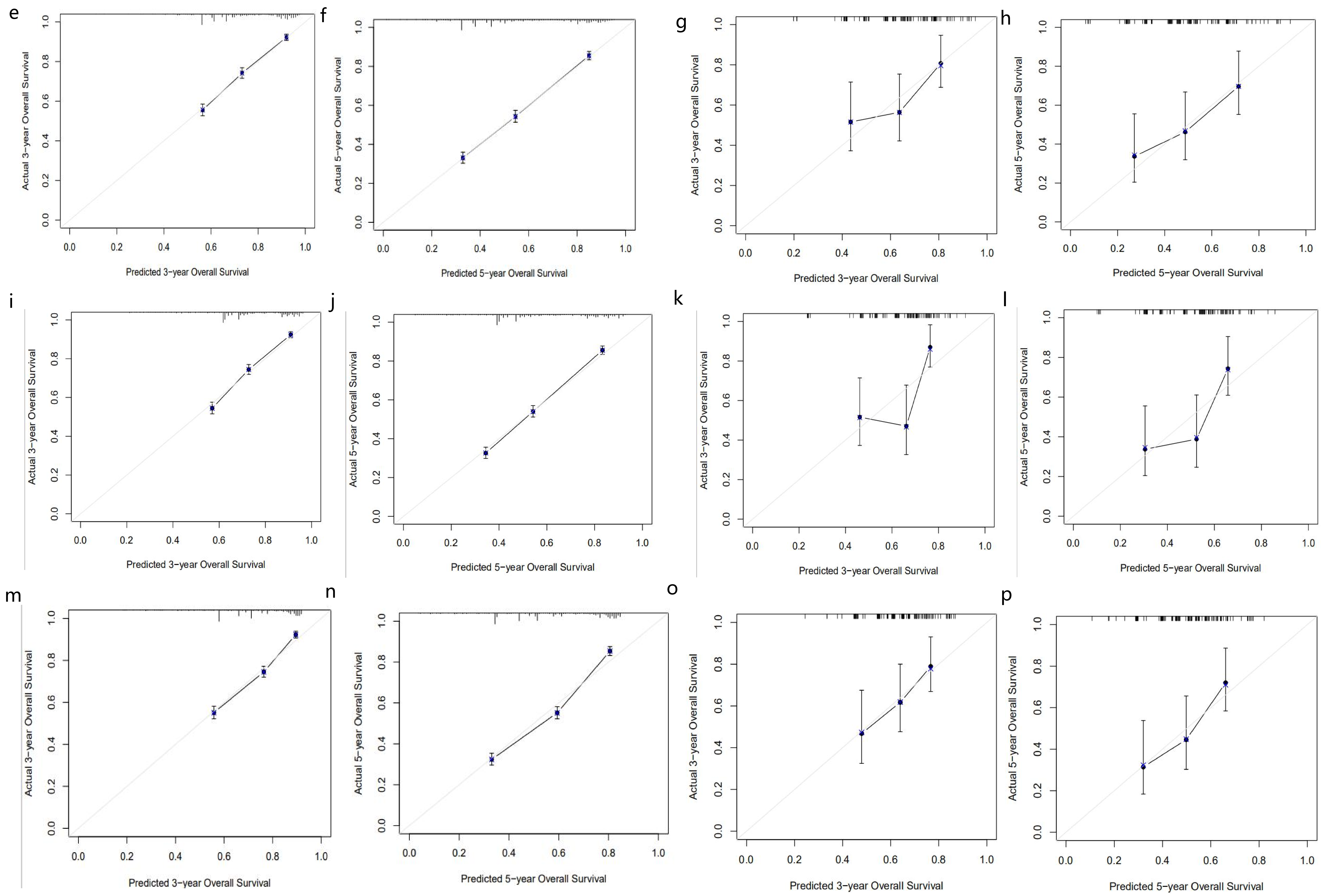

3 year and 5 year survival calibration curves for patients(e,f and g,h are Nomogram; i,j and k,l are DeepHit; m,n and o,p are RSF).
