# Supplementary material for: Predicting the prognosis of epithelial ovarian cancer patients based on deep learning models
Source: Front Oncol. 2025 Jul 25;15:1592746. doi: 10.3389/fonc.2025.1592746 (PMC12331489; doi:10.3389/fonc.2025.1592746)
Supplement: Supplementary file 4 [file DataSheet4.pdf]

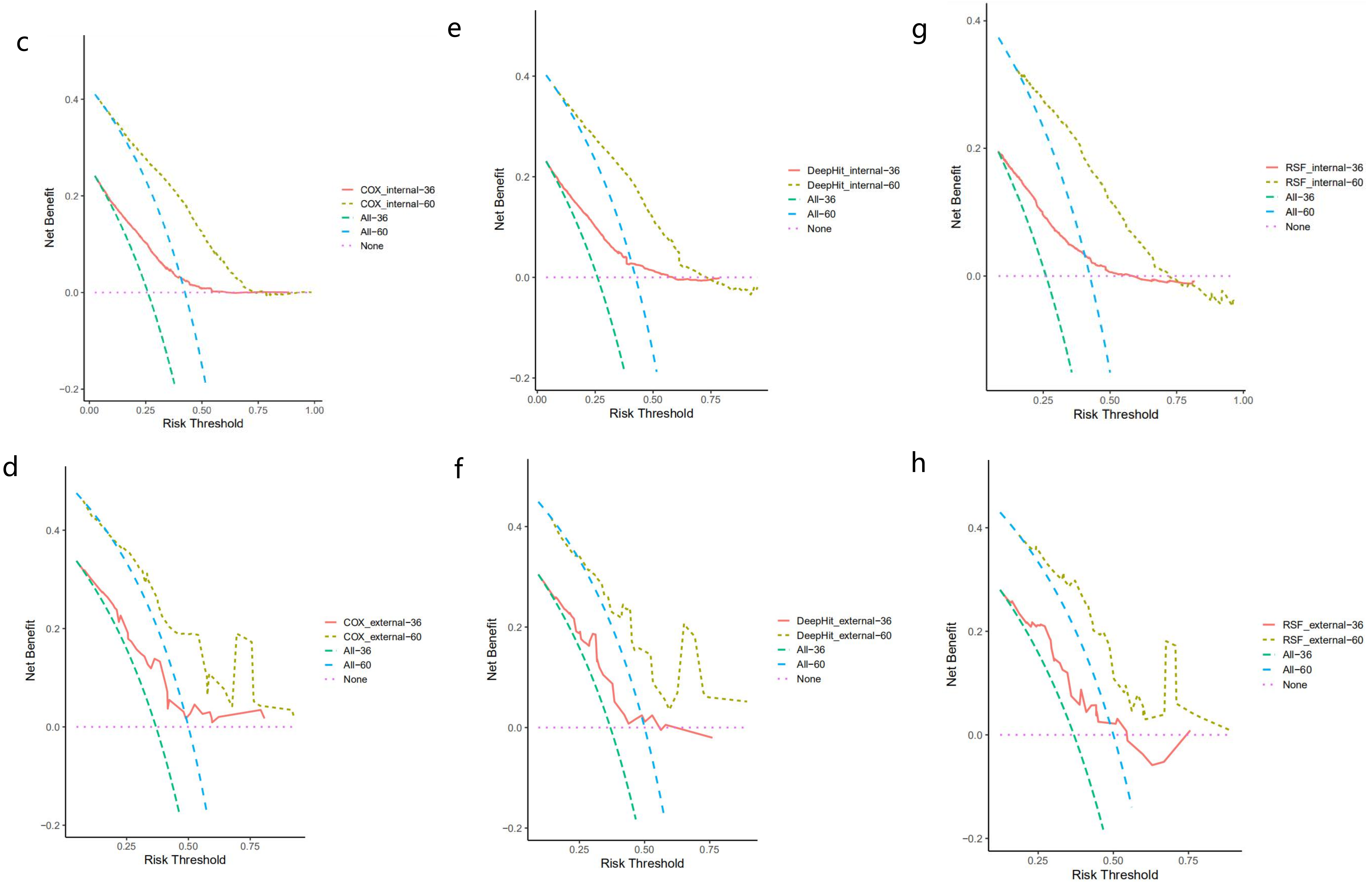

3 year and 5 year decision curve analysis for patients(c,e,g are internal validation set;d,f,h are external validation set.c and d are Nomogram; e and f are DeepHit; g and h are RSF).
